# Desert locust outbreaks: ecological and economic impacts and management strategies

**DOI:** 10.1186/s12983-026-00613-6

**Published:** 2026-05-06

**Authors:** Diriba Fufa Serdo, Zoltán Németh

**Affiliations:** 1 Department of Evolutionary Zoology and Human Biology, University of Debrecen, Debrecen, Hungary; 2 Department of Biology, Ambo University, Ambo, Ethiopia

**Keywords:** Desert locust, Early warning systems, Ecological and economic impacts, Food security, Integrated pest management, *Schistocerca gregaria*

## Abstract

The desert locust (*Schistocerca gregaria*) is the most destructive migratory pest in the world, threatening food security and livelihoods, particularly across Africa, the Middle East, and Southwest Asia. This review examines historical outbreak trends, emphasizing their cascading ecological and socio-economic impacts, which extend from acute agricultural losses to long-term environmental harm from chemical control. Despite advances in early warning systems, biopesticides, and integrated pest management (IPM) frameworks, their effectiveness is systematically undermined by a persistent implementation crisis. This crisis is characterized by a critical warning–response gap—where delays in funding and coordination mismatch the rapid pace of locust breeding—and by widespread failures to apply established environmental and safety protocols during emergency campaigns. Consequently, the central challenge of contemporary locust management lies not in a lack of technical knowledge, but in the governance deficit that prevents its application. We argue that achieving sustainable control requires a decisive shift in focus toward the structural strengthening of the financial, institutional, and cooperative frameworks essential for rapid, safe, and ecologically sound intervention. Ultimately, desert locust management must be explicitly embedded within broader agendas for climate adaptation, food systems resilience, and biodiversity conservation, supported by robust regional cooperation, inclusive community engagement, and cross-sectoral governance.

## Introduction

The desert locust, *Schistocerca gregaria* (Forskål,1775) is the most destructive migratory pest in the world known for forming large, mobile swarms. These swarms can travel long distances, causing severe agricultural devastation, food insecurity, and significant economic losses across the deserts and adjacent regions of Africa, the Arabian Peninsula, and Southwest Asia [1–4]. A key biological feature underpinning this threat is density-dependent phase polyphenism, where the species exhibits two distinct phenotypic forms: the solitarious and the gregarious phase [5, 6]. At low population densities, locusts remain in the solitarious phase—cryptic, sedentary, and avoiding conspecifics. Under conditions of crowding and altered resource availability, a behavioral and physiological transformation leads to the gregarious phase, characterized by brightly colored, highly active, and collectively migratory individuals [6–9].

It is important to note that while the gregarious phase is defined by its dense, diurnal swarms, solitarious individuals also possess a migratory capacity, primarily migrating at night [10, 11]. It is the mass, coordinated movement of the gregarious phase, however, that forms the large-scale destructive swarms. This capacity for gregarious swarming underpins the formal classification of locust population dynamics into escalating stages. An initial outbreak involves localized population increases and gregarization, often in an area of approximately 5,000 km² [2, 12]. Uncontrolled outbreaks can escalate into an upsurge, marked by substantial population growth over multiple generations and larger regions. If an upsurge remains unchecked and ecological conditions continue to support breeding, it may develop into a plague, which is marked by widespread and severe infestations affecting extensive regions [13]. A major plague is defined as one in which two or more regions are simultaneously affected [2]. Progression through these stages is tightly linked to climate variability, which influences the rainfall, temperature, and vegetation patterns critical for locust breeding and survival [14, 15].

Research on the desert locust has generated a substantial body of literature, yet key integrative gaps persist. Existing syntheses often provide deep but compartmentalized insights, analyzing climatic drivers, historical outbreak trends, socio-economic impacts, or control strategies in relative isolation. However, there is a critical need for a synthesis that integrates these domains to holistically analyze outbreak dynamics, from cause to consequence to management. Furthermore, rapid advances in forecasting and management strategies require contemporary appraisal. Therefore, this review aims to provide a comprehensive analysis by: (1) synthesizing long-term historical patterns of desert locust outbreaks within the context of climate variability; (2) assessing their multi-faceted ecological and economic impacts; (3) evaluating current and emerging management and forecasting strategies; and (4) analyzing the barriers that prevent the full implementation of established safety and management frameworks.

## Historical trends, geographical distribution, and major drivers

### Historical records and outbreaks of the desert locust

The documented history of desert locusts spans millennia, with references to their damage dating to Assyrian times (~ 3200 BC) and in ancient texts, underscoring their long-standing societal impact [16–18]. However, these historical records are primarily qualitative, lacking a systematic, mechanistic synthesis that links past outbreak patterns to environmental triggers. Historically, gregarious outbreaks have occurred sporadically over millennia, typically originating in well-known but isolated breeding regions [1, 19]. The literature details major 20th century plagues from 1926–1934,1940–1948, 1949–1963, 1967–1969 [12], and 1986–1989 [1, 20]. The progression from a localized outbreak to a continental plague is intrinsically tied to environmental sequences across complementary seasonal breeding areas [2, 13]. Crucially, most significant breeding sites are situated in challenging terrains frequently affected by insecurity, including armed conflict and landmines, which preemptively hinder monitoring and response [1, 19, 21–23].

The interplay of climate and instability becomes starkly visible in the post-1990 record. The upsurges from 1992 to 1994 [24–26] and 2003–2005 [25, 27, 28] along with the 1998 outbreak [29], were contained relative to plagues. During 2005–2018, there were no plagues of desert locusts; however, several localized outbreaks—including events in Ethiopia and Saudi Arabia in 2008 and widespread activity from Mauritania to the Near East from 2012 to 2013—did occur, leading to swarm formation [9]. Activity persisted along the Red Sea and Horn of Africa until 2015, with variable risk continuing thereafter in Yemen, Saudi Arabia, Sudan, and Mauritania. This precarious balance was severely disrupted during the 2018–2021 desert locust upsurge, one of the most severe in recent history. The chain of events began with a clear environmental trigger: heavy rains from two tropical cyclones in the Arabian Peninsula in late 2018 [30]. However, its unprecedented severity is widely attributed to concurrent conflicts in the Middle East and Eastern Africa that critically disrupted control capacities [22, 30]. The initial outbreak escaped control due to unpreparedness in Saudi Arabia, and the impacts of armed conflict in Somalia and Yemen, which crippled surveillance and control, contributing to the invasion of ≥ 22 countries [19]. This event crystallizes the modern driver framework: climate variability, particularly extreme rainfall events, provides the initial catalyst, but socio-political instability is the decisive exacerbating factor that can transform an upsurge into a full-blown plague.

The literature provides effective diagnosis of past outbreaks but lacks a comprehensive forward-looking synthesis. While 20th century efforts by the Food and Agriculture Organization (FAO) and others established management strategies focused on hotspot identification and control [2, 16, 30, 31], the field has yet to fully integrate historical lessons into models projecting the broader implications of climate change. The critical gap is not in documenting history, but in mechanistically synthesizing it to forecast how the combined effects of climate change, socio-political instability, and institutional barriers will redefine the locust threat in the 21st century—a forecast that requires a detailed understanding of the species’ climatic sensitivities.

### Geographical distribution and climatic drivers

Desert locusts inhabit the arid and semi-arid zones of Africa, the Middle East, and Southwest Asia. During quiet periods (recessions), their solitarious populations are typically restricted to a vast recession area estimated at between 16 million and 29 million km², where annual rainfall is less than 200 mm [9]; (FAO: http://www.fao.org/locusts/en/; accessed on 5 November 2024). However, during upsurges and plagues, swarms can invade an expanded area of up to 32 million km², demonstrating high-mobility and a metapopulation structure where seasonal breeding zones are interconnected by long-distance swarm flights of up to 150 km per day [2]. Remote sensing has identified persistent invasion hotspots, such as Morocco, the Sahel (parts of Mauritania/Senegal to Sudan), northern Ethiopia, South Sudan, northwestern Kenya, the southern Arabian Peninsula, and parts of Pakistan and India [32].

The species’ distribution and impact are dictated less by fixed boundaries than by the interaction of its life cycle with specific climatic parameters, a relationship now under threat of alteration by climate change. The desert locust lifecycle is critically dependent on specific climatic conditions, with each stage (egg, nymph, adult) governed by precise thresholds that dictate population survival and growth. Successful reproduction is contingent on soil moisture (5–25%) and temperature (15–35 °C), which allow eggs to absorb sufficient water—approximately their own weight—necessary for hatching [33–36]. Heavy rainfall, particularly from tropical cyclones, creates ideal egg-laying habitats and drives rapid population growth [32]. Conversely, extended droughts and high temperatures deter egg-laying, as locusts avoid overly dry or excessively hot soils that do not meet their moisture requirements [37]. However, the sensitivity of egg mortality to sub-optimal conditions—such as temperatures exceeding 35 °C, flooding, or specific moisture deficits—remains quantitatively underexplored [12], introducing uncertainty into projections of climate change impacts.

Nymph survival and development are primarily influenced by temperature and vegetation quality. Development rates accelerate with rising temperatures, while extreme weather events that spur rapid vegetation growth can enhance survival [12, 32]. Yet, the literature does not sufficiently detail how temperature deviations outside observed ranges or prolonged drought directly affect nymph mortality rates, constraining the reliability of predictive models. Adult movement, the engine of invasion, is governed by key meteorological triggers. Solitarious adults migrate at night when temperatures exceed 20–22 °C with wind speeds below 6 m/s. Swarm take-off depends on solar radiation and temperature (15–17 °C in sun, 23–26 °C in cloud) and is inhibited by high winds > 6 m/s [12]. While these relationships are established, the influence of increasingly frequent and intense extreme wind events—a predicted consequence of climate change—on migratory disruption remains understudied.

### Climate interactions and critical knowledge gaps

The prevailing dynamic is one of climate variability: extended droughts suppress populations, but subsequent heavy rainfall creates ephemeral, optimal conditions for explosive breeding [30, 38]. Climate change acts as a threat multiplier by potentially altering the frequency, intensity, and geographic pattern of these triggering rainfall events [3, 39, 40]. This interaction is further complicated by human factors, as agriculture near desert margins expands favorable habitat, and land-use changes alter local microclimates and vegetation [2, 41]. While the recent development of advanced mechanistic models has resolved core questions of life-stage sensitivity to temperature [42] and rainfall-driven phase dynamics [43], critical synthetic gaps persist at the scale necessary for forecasting and management. First, evidence on future geographic distribution under climate change remains inconclusive, with projections of recession range contraction and marginal expansion lacking certainty [44]. This macro-scale biogeographic uncertainty presents a fundamental limit to long-term risk mapping. Second, and more critically, a chasm remains between these biophysical models and the operational frameworks needed for decision-making. There is a pressing need for a translational synthesis that dynamically couples the insights from mechanistic population models with continental-scale atmospheric dynamics, explicit projections of land-use change, and—most importantly—models of human socio-political vulnerability and institutional capacity.

## The ecological and economic impacts of desert locusts

The desert locust poses a significant threat to global food security and economic stability, inflicting widespread devastation on agricultural crops, pasturelands, and fodder resources [1]. Its impacts are characterized by devastating scale and cascading consequences. A single swarm of 1 km², containing up to 80 million locusts, can consume the equivalent daily food of 35,000 people [45, 46]. Operating within a potential invasion zone across parts of Africa and Asia, locusts directly threaten the livelihoods of approximately one-tenth of the global population [2, 9, 47]. The resulting economic and ecological damage is profound, multifaceted, and often persistent.

### The multifaceted economic impact of desert locust crises

The economic and agricultural impacts are severe, encompassing both direct asset destruction and the immense, recurrent costs of control. Historical records quantify this devastation. These include the destruction of 1 million grapevines (*Vitis vinifera*) in Libya in 1941, an estimated USD 50 million in losses during a single growing season in Morocco from 1954 to 1955 [1], and a staggering loss of 167,000 tons of grain in Ethiopia in 1958, which was sufficient to feed 1 million people for one year [1, 48]. Moreover, the 2003–2005 upsurge affected over 8 million people across 20 countries, caused an estimated USD 2.5 billion in crop losses, and required the pesticide treatment of 13 million hectares at a cost exceeding USD 500 million [1, 9, 27, 28, 49]. According to FAO, the affected regions in Sub-Saharan Africa experienced crop losses ranging from 80% to 100%, and the costs associated with control efforts exceeded USD 500 million [28, 49]. These figures, however, often fail to capture the full economic burden, which includes market destabilization, trade disruptions, and long-term impacts on agricultural investment and gross domestic product (GDP), the cascading societal costs of food aid and education.

**Fig. 1:**
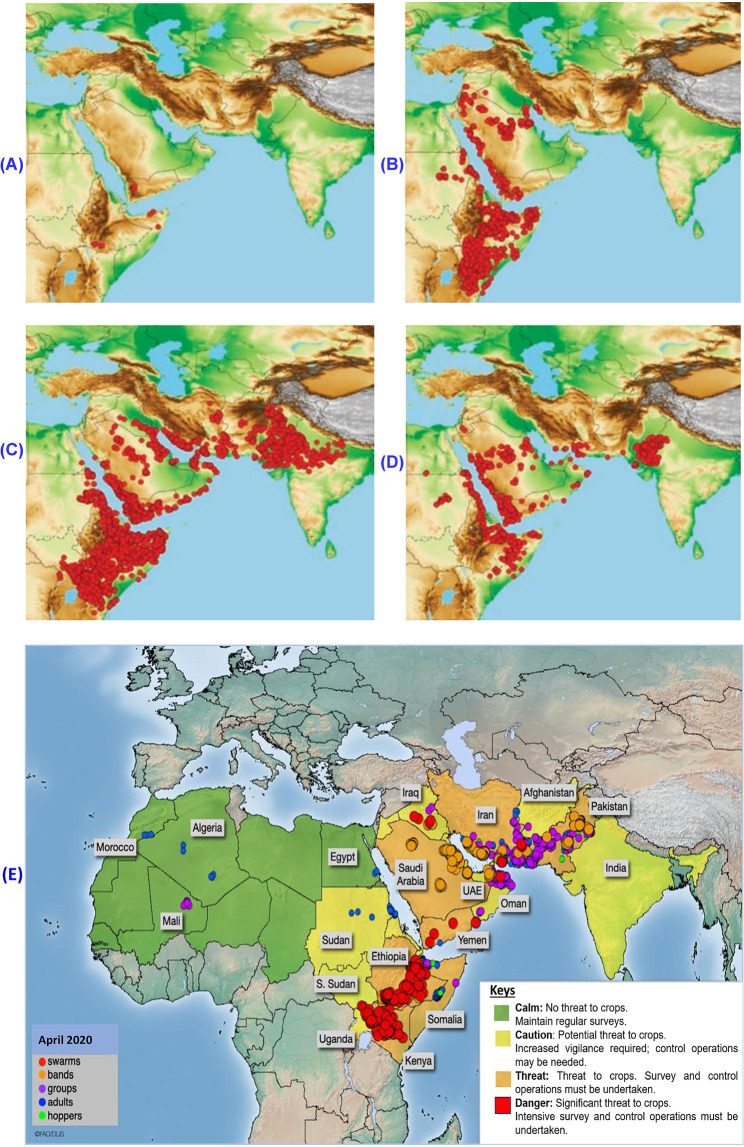
Rapid geographic expansion of the 2018–2021 desert locust upsurge. (**A**) Undetected breeding begins in the Arabian Peninsula following cyclonic rainfall in 2018. (**B**) Initial swarm concentrations form and begin spreading from the Arabian Peninsula to the Horn of Africa and Southwest Asia in 2019. (**C**) Dramatic large-scale spread by April 2020, with widespread infestations across the Horn of Africa, the Arabian Peninsula, and Southwest Asia. (**D**) Continued monitoring and mop-up operations as the upsurge declines, with residual activity in the Horn of Africa and Yemen. (**E**) Labelled map showing major invasion pathways and regional hotspots during 2020 phase of the upsurge [45]

The 2018–2021 desert locust upsurge, one of the most severe in recent history, underscored the escalating and transboundary nature of the threat [34]. Characterized by an unprecedented rate of geographic spread, the crisis originated with outbreaks in the Arabian Peninsula remote interior Rub al Khali in late 2018 [22]. It then escalated rapidly, affecting large areas of Eastern Africa, the Red Sea region, and Southwest Asia by the first half of 2020, as shown in Fig. 1. This rapid expansion overwhelmed national monitoring and control capacities, directly translating into severe on-ground impacts. The first wave in late 2019 destroyed approximately 70,000 hectares of cropland in Somalia and Ethiopia and 2,400 km² of pasture in Kenya. By mid-2020, the crisis intensified, with Ethiopia alone reporting catastrophic damage to 114,000 hectares of sorghum, 41,000 hectares of maize, and 36,000 hectares of wheat, triggering massive cereal losses and sharp price inflation [50].

Moreover, a national assessment in Pakistan projected crop losses of USD 1.2–2.9 billion across 61 districts, devastating staple crops and reducing agricultural productivity—a sector constituting 20% of national GDP—by an estimated 2% in 2020 [1]. The international counter-offensive was unprecedented: the FAO-coordinated response treated over 760,000 hectares by mid-2020, averting USD 456 million in cereal losses [51]. By the upsurge’s conclusion, 2.29 million hectares had been treated (2020–2021), preventing an estimated USD 1.77 billion in crop and milk losses and protecting 41.5 million people from acute food insecurity [45]. Overall, more than 38,000 control operations were deployed across nearly 50 countries [3, 52, 53]. These impacts transcend agriculture, directly exacerbating malnutrition, health risks, and poverty—especially among vulnerable groups—and creating a vicious cycle where outbreaks deepen food insecurity and erode community resilience to future shocks [54, 55].

### Ecological impacts: a dual threat from pest and pesticide

The ecological impacts of desert locust infestations are dualistic, stemming from the pest itself and the primary means of its control. The primary impact is rapid and severe ecosystem degradation caused by intensive herbivory. Swarms induce widespread defoliation of both cultivated and native vegetation, leading to immediate habitat loss for other species, reduced ground cover, and accelerated soil erosion. This not only shatters food systems but also destabilizes ecological communities and critical ecosystem functions, such as water regulation and soil fertility [50, 56]. The second, the broad-spectrum chemical insecticides used for control inflict substantial collateral damage. The use of non-selective chemicals, while necessary for rapid swarm suppression, cause significant environmental damage by harming beneficial insect populations, including pollinators and natural pest predators, and pose direct risks to avian and aquatic species through contamination [57, 58].

The risks of pesticide use are well-understood, and detailed international guidelines exist to mitigate them. The FAO’s Desert Locust Guidelines [59] provide a comprehensive framework for risk reduction, mandating specific protocols for insecticide selection (based on WHO/GHS hazard classifications), the application of buffer zones, the use of personal protective equipment, and the safe management of waste and empty containers. However, the immense scale of chemical use during upsurges and plagues, often under crisis conditions, means these protocols are frequently compromised. This is illustrated by the 2020–2021 campaign, which applied 710,000 L of conventional pesticide and 2,760 kg of biopesticide across ten countries in eight months [51]. This aggressive response creates a critical paradox: it addresses an immediate ecological crisis while precipitating longer-term environmental and public health risks from pesticide residues—risks that are magnified when logistical and financial constraints prevent full implementation of safety protocols. Furthermore, the post-campaign legacy constitutes a second implementation crisis, involving the formidable challenges of safe disposal for thousands of empty containers, bioremediation of contaminated sites, and management of leftover pesticide stocks [45].

## Strategies for sustainable desert locust management

Effective management operates within a clear hierarchy of strategic goals: reaction, proaction, and outbreak prevention. Reaction is the costly, defensive protection of crops during plagues. Proaction, the current best practice, involves intervening during localized outbreaks to prevent escalation into upsurges. Outbreak prevention, the ultimate goal, aims to suppress the very onset of gregarization, a strategy largely unrealized since the banning of persistent organochlorine pesticides [19]. Contemporary Integrated Pest Management (IPM) frameworks primarily operationalize the proactive approach while research aims to rebuild capacity for true prevention.

### Monitoring and early warning systems

The cornerstone of sustainable locust management is an effective early warning system, which integrates technological prediction with sustained human field capacity for a coordinated response. This effort is fundamentally supported by the FAO through its Locust Group and Desert Locust Information Service (DLIS), which provides critical risk information and facilitates collaboration among affected countries [60–62]. The foundation of this system has long been field surveillance, conducted by national locust control centers, though these surveys are often challenged by the vast and remote nature of locust habitats [9].

Remote sensing technology has been developed to augment these ground-based efforts. The use of satellites has proven effective for the surveying of desert locust [63]. The development of the Normalized Difference Vegetation Index (NDVI) enabled the identification of green vegetation in arid regions, with applications across Mauritania, Sudan, and Mali [9]. The integration of Moderate Resolution Imaging Spectroradiometer (MODIS) data for rainfall estimation and tools like the RAMSES GIS platform has significantly enhanced habitat assessment and data management [64]. However, significant limitations persist, as coarse-resolution NDVI (e.g., 8 km) is often a poor predictor of localized breeding sites [65]. Predictive accuracy is only robust when remote sensing is combined with ground-truthing—historical survey data, field observations of soil moisture, and post-rainfall vegetation [48, 66, 67].

This necessity for integrated data has driven the transition from descriptive mapping to predictive, dynamic forecasting. The 2018–2021 upsurge acted as a catalyst for rapid digital innovation within FAO’s early warning system, culminating in tools like eLocust3m (smartphone app), eLocust3g (satellite GPS), and the EarthRanger digital control room. These together facilitated over 135,000 field reports in 2021 and aimed to optimize aerial asset deployment [45]. Parallel institutional efforts, such as the open-access Locust Hub platform, modernize data analysis and sharing, while emerging frameworks integrate remote sensing with life-cycle and atmospheric models to simulate swarm dispersal [68]. Therefore, the state of the art is a hybrid system. Its reliability is not a function of technology alone but of the sustained human capacity to conduct dangerous and logistically challenging field reconnaissance, which validates and refines the technological predictions.

### Control strategies: efficacy, trade-offs, and technological horizons

#### Chemical control

Chemical control remains the primary tool for emergency suppression of infestations of many bands and of large swarms. Its historical evolution reflects a growing—albeit costly—understanding of ecological trade-offs. The highly persistent organochlorine dieldrin was the cornerstone of mid-20th century control as it facilitated barrier treatments against hopper bands but was globally banned due to its environmental and health impacts [69, 70]. Current international standards, as outlined in the FAO’s operational and safety guidelines [59], emphasize a risk-managed approach. This includes the selection of faster-degrading compounds applied via ultra-low-volume (ULV) formulations to reduce total chemical load, as recommended by the FAO Pesticide Referee Group. Crucially, the FAO guidelines establish a structured framework for the entire control campaign, from pre-campaign medical preparation and training to post-campaign waste disposal, aimed at minimizing occupational and environmental risk [59].

The scale of pesticide use during major plagues is immense, with millions of liters applied over vast areas [46, 71]. While highly efficacious, their broad-spectrum nature causes significant collateral damage to beneficial insects, pollinators, and natural predators, creating a paradox where control efforts can undermine long-term ecological resilience [57, 58]. The effective implementation of safety guidelines, including the deployment of specialized monitoring teams to assess spray quality, occupational health, and environmental side-effects, is essential to managing this paradox [59]. The potential for insecticide resistance warrants consideration. The desert locust’s biology—specifically its limited annual generations and highly migratory behavior—may reduce the likelihood of resistance development compared to pests with more continuous, localized populations. Published reports of confirmed resistance in *Schistocerca gregaria* field populations are lacking. Nevertheless, the reliance on a limited arsenal of chemical classes underscores the importance of proactive resistance monitoring, particularly as control efforts intensify during upsurges, and highlights the need for continued investment in insecticides with diverse and novel modes of action.

Beyond chemical options, emerging tools such as drones (UAVs) are being integrated not primarily for large-scale spraying, but as surveillance force multipliers to guide targeted interventions [72]. However, their operational capacity for widespread control remains limited by small payloads, brief flight endurance, and high costs [70]. Critically, as [73] argue, the deployment of any advanced technology is often secondary to chronic human and institutional constraints. The primary impediment is therefore not a lack of tools, but systemic human and institutional constraints that limit effective deployment and adherence to established safety protocols—even when those protocols exist.

#### Biological control

In the face of increasing restrictions on many chemical pesticides, biological control has become an important cornerstone of modern, ecologically sustainable pest management. A prominent example is the use of the entomopathogenic fungus *Metarhizium acridum*, which is highly specific to acridids—including desert locusts—and poses minimal risk to non-target organisms. Its efficacy has been demonstrated in large-scale field applications [13, 74]. Commercialized as Green Muscle^®^, Green Guard^®^, and Novacrid^®^, it has been deployed operationally from Australia to East Africa. In Australia, Green Guard^®^ was used on over 100,000 hectares between 2000 and 2010 for preventive control of the Australian plague locust [75]. Its key advantages are its environmental safety, a lack of pesticide resistance issues, and its compatibility with ecosystem function—for instance, birds can safely consume infected locusts [70, 76, 77].

The microsporidian *Nosema locustae* (also known as *Paranosema locustae*) is another well-established agent, particularly in China and North America. It offers unique benefits: long-term persistence in host populations for over a decade, vertical transmission to offspring, and a broad host spectrum within Orthoptera (> 144 species) [31, 78]. Beyond direct mortality, *P. locustae* induces significant sublethal effects that disrupt locust population dynamics. For example, in the migratory locust (*Locusta migratoria*), infection with *P. locustae* has been shown to reduce levels of the neurotransmitter taurine, promoting a shift in morphology and behavior towards the solitarious phase [79]. This illustrates a potential mechanistic pathway through which such biocontrol agents can disrupt the gregarization process central to plague formation.

However, the major bottleneck for *M. acridum* use is the high cost and complexity of in vivo mass production, which limits its scale and economic viability for emergency responses. Furthermore, its interaction with other biocontrol agents is nuanced. While laboratory studies show that combined application with *Metarhizium anisopliae* can have additive or synergistic effects on host mortality, this comes with a critical trade-off: concurrent fungal infection severely reduces the production of *N. locustae* spores within the host cadaver, potentially undermining the very long-term, recyclable pest suppression that is its key advantage [80]. The universal limitation of biopesticides is their slower speed of kill (5–21 days for peak mortality) compared to chemicals, making them less suitable for rapid crisis response but ideal for strategic, preventive treatment in known breeding areas as part of an integrated strategy.

#### Physical and mechanical control

Physical and mechanical methods are best suited for low-density infestations, community-led actions, or as components of habitat management. The primary approach involves habitat manipulation to disrupt the locusts’ breeding and feeding cycles. This includes removing vegetation that provides food and shelter and plowing fields to destroy egg pods and breeding habitats. Beyond habitat alteration, direct interventions such as the nocturnal burning of roosting locusts, digging trenches to trap and bury hoppers, and deploying barriers to intercept advancing bands are also employed [62]. However, these methods share profound limitations that restrict their role in modern management. They are highly labor-intensive and logistically challenging to implement, a critical flaw given that locust outbreaks often originate in remote, inaccessible regions with few inhabitants. When large-scale swarms invade agricultural zones, their immense density and mobility swiftly overwhelm such manual efforts. Ecologically, they can be counterproductive: fire damages native flora and fauna, and trenching promotes soil erosion. A related strategy is the harvesting of desert locusts for use as food or feed. While this offers a potential benefit, it is not a reliable control method and requires stringent safety protocols to manage risks from the presence of pesticides or pathogens in collected locusts [81–83]. Therefore, physical controls are of limited value within the large-scale, transboundary framework required for plague prevention, though they may have localized or cultural relevance.

#### Pheromone and genetic approaches

Pheromone and genetic tools represent next-generation approaches, promising high specificity in locust control. However, they remain largely in the research phase, illustrating the significant gap between technological potential and field-ready application.

**Pheromone Control**: Research over decades has identified various pheromones influencing aggregation, maturation, and courtship, such as phenylacetonitrile (PAN), which acts as an inhibitor or repellent [84–86]. Despite these behavioral insights, no volatile pheromone has demonstrated substantial utility in practical field control. The transition from laboratory discovery to a reliable, scalable control technology remains a persistent and unmet challenge, but an ongoing need.

**Genetic Approaches**: The sequencing of the *S. gregaria* genome [87] has opened avenues for genetic pest management. In laboratory settings, RNA interference (RNAi) has shown promise for silencing vital genes related to digestion and reproduction via ingested double-stranded RNA (dsRNA) [88, 89]. However, major barriers to field deployment persist, including dsRNA instability, the high cost of production, and the difficulty of ensuring efficient oral delivery to locust populations in the wild [88, 90, 91]. More recent techniques, such as CRISPR/Cas9 gene editing, present even more profound possibilities and complexities, though they remain at an even earlier stage of development for this species. Importantly, advances in these strategies for other insect pests—particularly various mosquito species—suggest a pathway for translation to locusts. Nevertheless, the very sophistication of these genetic and pheromone tools underscores a central dilemma highlighted by [73]: the chasm between technological promise and operational reality. Notably, recent advances in dsRNA production and application for other agricultural pests—such as the first sprayable dsRNA biopesticide, Ledprona, developed for the Colorado potato beetle (*Leptinotarsa decemlineata*)—demonstrate that scalable production and regulatory pathways are achievable [92]. As with the development of fungal biopesticides, bridging this chasm will require targeted R&D investment to optimize production and efficacy, alongside regulatory frameworks adapted to the unique characteristics of these new technologies.

### Towards Integrated Pest Management (IPM)

**Fig. 2:**
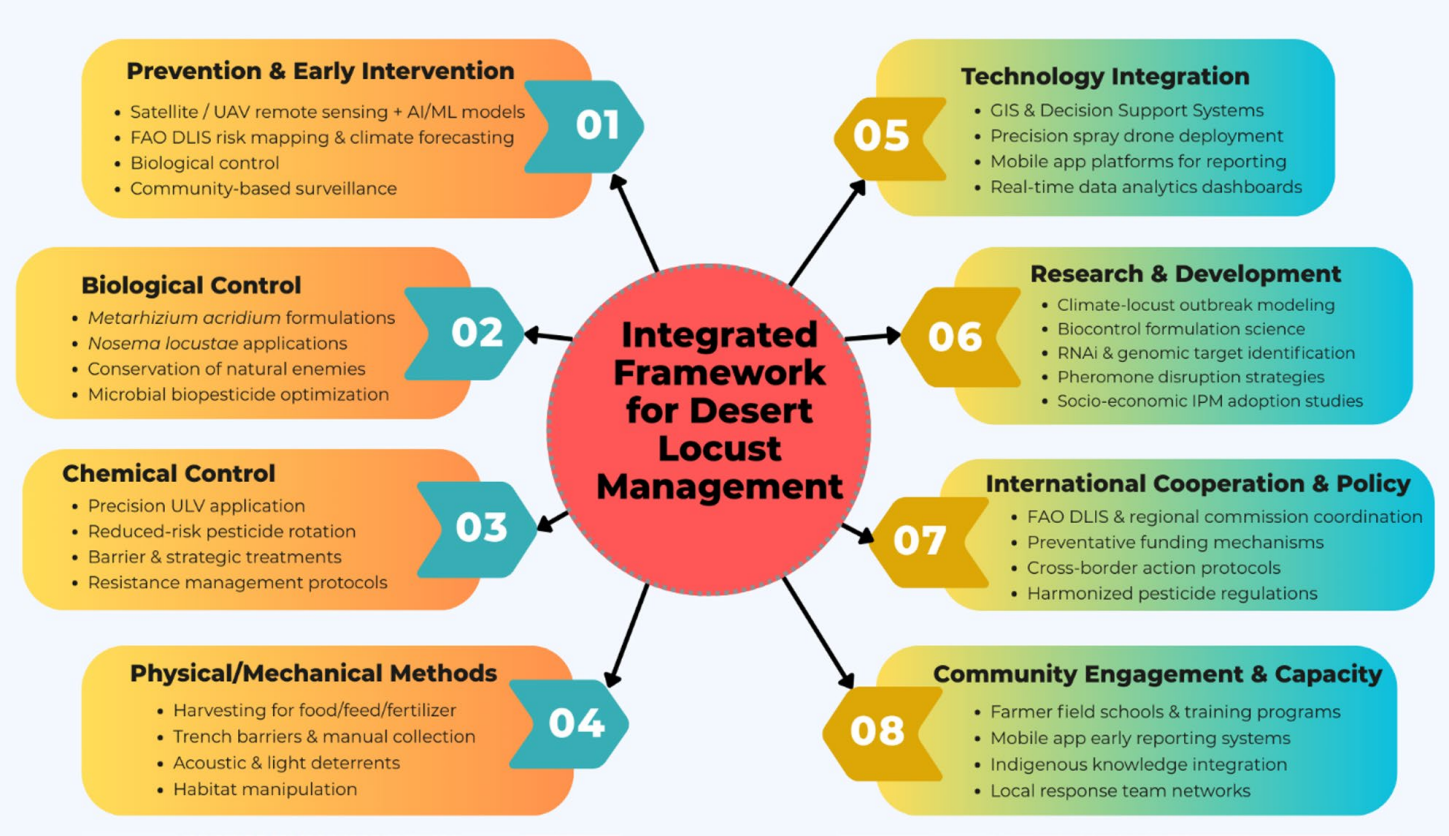
Integrated framework for desert locust management. The framework integrates eight core components within an adaptive cycle. Operational interventions (left or 01–04) are enabled by systemic pillars (right or 05–08), with data-driven monitoring at its foundation

IPM for desert locusts is a holistic strategy that dynamically combines the above tools based on the population phase and context, aiming to maximize efficacy while minimizing economic, environmental, and health costs. As shown in Fig. 2, a functional IPM framework operates on a prevention-to-response continuum, underpinned by a global early warning system coordinated by the FAO’s DLIS. This technical framework is supported by a parallel procedural framework for safety and environmental protection [59]. The strategic application follows a gradient aligned with outbreak intensity. The cornerstone is prevention & early intervention during recession phases, which leverages early warning to target areas for pre-emptive suppression using biological control and habitat management. The critical importance—and vulnerability—of this phase was starkly illustrated during the 2003–2005 West Africa upsurge, where accurate FAO warnings were rendered ineffective by a critically slow international donor response, creating a fatal “warning-response gap” [25]. The quantitative logic of this preventive approach is increasingly supported by integrated dynamical models, which demonstrate that coordinated interventions achieving 20–40% efficacy in targeting key lifecycle stages can suppress potential plague-scale infestations to recession levels [43]. This provides a crucial benchmark for the operational goals of proactive management, underscoring that even modest, well-timed investments in early action can yield disproportionately large returns in crisis aversion.

During a contained outbreak response (upsurge phase), localized population growth is managed with a combination of biological control and targeted, reduced-risk chemical insecticides. At this stage, operational safety protocols, such as the use of pesticide use passports for applicators and cholinesterase monitoring for organophosphate exposure, become critical components of responsible management [59]. Finally, during an emergency plague response, the rapid deployment of chemical control remains unavoidable to safeguard food security. Here, the IPM challenge is greatest: ensuring chemical use is precise and adheres to stringent environmental safeguards, such as respecting buffer zones around sensitive areas and enforcing livestock withholding periods, as mandated by FAO guidelines. This IPM framework must also contend with powerful external drivers like climate change and landscape change [93]. Therefore, a robust IPM system requires more than technical components; its success is contingent on establishing resilient monitoring networks, aligning warning systems with local practices, empowering communities, and fostering the collaborative, cross-disciplinary efforts necessary to build the sustained institutional capacity required to implement both control and safety protocols effectively.

## The implementation crisis: barriers to effective desert locust control

Modern desert locust management relies on advanced early warning systems and integrated control strategies. Despite these technological and strategic advances, a persistent gap remains between predictive capability and operational effectiveness. Research continues to produce advanced forecasting models that link climate data with locust population dynamics [42, 43]. Yet the predictive power of these tools is often undermined by socio-political and institutional constraints. This systemic difficulty—the failure to translate technical knowledge into effective action—defines the implementation crisis. The synthesis reveals that, while bioclimatic factors determine the initiation and potential scale of outbreaks, human and institutional factors increasingly govern their actual trajectory and ultimate socio-economic impact.

This crisis is fundamentally crystallized in the chronic “warning-response gap,” where accurate forecasts fail to trigger timely action. As demonstrated during the 2003–2005 upsurge, slow donor response transformed accurate forecasts into a USD 500 million crisis [25, 28]. This gap typically manifests not as a failure of surveillance, but as a proaction gap where detected outbreaks are not controlled swiftly, allowing escalation [19]. Empirical evidence underscores the dominance of these socio-political barriers over technical ones. A 35-year review of 13 major breeding countries found that direct insecurity (armed conflict, terrorism, landmines) affected 77% of nations during major plague years, with the indirect effects of conflict—resource diversion and institutional erosion—being even more pervasive [22]. Similarly, a survey during Kenya’s 2018–2021 upsurge quantified that 70% of constraints were human-resource and institutional limitations (e.g., political obstacles, coordination failures), while technological challenges accounted for only 15% [73, 94]. Thus, the primary constraint is no longer technical understanding, but the governance, coordination, and financing required for effective implementation. The implementation crisis constitutes the decisive bottleneck through which bioclimatic risk is transformed into human catastrophe.

National case studies illustrate how these systemic barriers manifest. Pakistan’s experience during the 2018–2021 episode exemplifies unpreparedness driven by institutional decay. A 27-year recession resulted in most equipment being obsolete or non-functional, and there being few trained locust workers able to respond quickly and implement a widespread treatment program [95]. In addition, response was hindered by geopolitical tensions that initially stalled bi-national coordination with India and Iran [1]. As a result, pre-emptive mobilization failed, despite FAO warnings of high risk in border deserts. Ultimately the control of the upsurge was a success, following a declaration of an emergency by the Government of Pakistan [96], but the initial slow response clearly illustrates the warning-response gap. The scale of the 2020–2021 upsurge itself was fueled by locust “engines” breeding unchecked in the chronically insecure zones of Somalia and Yemen, from where swarms reinvaded controlled areas [1, 22].

Even when technical solutions are available, their implementation is hindered by infrastructural deficits and regulatory misalignment. Advancements in monitoring tools like UAVs face practical deployment challenges [70]. Furthermore, the development of next-generation tools (e.g., RNAi, transgenics, pheromones) is hindered by the need for sustained research investment and navigation of intricate biosafety regulations—processes misaligned with the urgent, crisis-driven nature of locust emergencies. The systemic failure to implement established safety and environmental protocols provides a stark measure of the implementation crisis. The comprehensive FAO Environmental, Health and Safety (EHS) standards prescribe a detailed “risk reduction process” spanning the entire campaign lifecycle, yet their execution is persistently undermined by underfunding and institutional fragility. For instance, the massive Pakistan response relied on chemical applications over nearly 300,000 hectares [1], highlighting the tension between emergency-scale intervention and safety adherence. This consistent inability to fully execute mandated protocols reveals the core problem: a sophisticated safety blueprint is ineffective without the sustained capacity to enact it. Therefore, while the scientific and technical blueprint for effective desert locust management is well-established [31, 59], its implementation remains precarious. Success hinges not on better technology alone, but on resolving the deep-seated human, political, and financial challenges that determine whether the paradigms of prevention, proaction, and safety can be realized.

## Conclusion and future prospects

This review establishes that the threat of desert locust plagues is a compound risk, arising from the convergence of precise bioclimatic triggers and chronic human system failures. While effective early warning tools, management strategies, and environmental safety protocols are well-documented, their impact is consistently undermined by a critical implementation gap. At times this gap is a result of armed conflict but more often this gap is manifested as a critical “warning-response gap” where chronic under-resourcing in key breeding regions allows controllable outbreaks to escalate into continental emergencies. The resulting impacts are dualistic. The immediate crisis leads to severe agricultural loss and food insecurity. However, the long-term consequences often include significant environmental and health damage from the large-scale application of broad-spectrum pesticides—a paradox where the control measure itself creates a secondary crisis, especially when safety guidelines are incompletely implemented.

Therefore, the central challenge of modern locust management lies less in a shortage of technical knowledge than in a systemic deficit of governance and institutional capacity. Progress requires coordinated advances with a renewed emphasis on implementation. A cohesive strategic agenda should be built on five pillars: (1) financial systems that enable rapid, pre-approved funding for early action; (2) an institutional mindset that reconceptualizes monitoring, response, and safety capacity as critical public infrastructure deserving of sustained investment; (3) scientific translation focused on converting insights from advanced mechanistic population models into practical, stage-specific intervention protocols for resource-limited settings and integrating them with models of socio-political vulnerability; (4) operational frameworks that mainstream EHS standards as a core indicator of programmatic success; and (5) strategic policy that explicitly integrates locust management into broader frameworks for climate adaptation, food security, and biodiversity conservation.

## Data Availability

No datasets were generated or analysed during the current study.
